# Epidemiological Insights into Carbapenem-Resistant *Enterobacterales* Throughout the COVID-19 Pandemic in Buenos Aires, Argentina

**DOI:** 10.3390/antibiotics15030273

**Published:** 2026-03-06

**Authors:** Francisco González-Espinosa, Francisco Magariños, Sofía Ciminello, Roque Figueroa-Espinosa, María Sol Haim, Tomas Poklepovich, Nicolas Potente, Cecilia Ormazabal, Gabriel Gutkind, Daniela Cejas, Marcela Radice

**Affiliations:** 1 Universidad de Buenos Aires, Facultad de Farmacia y Bioquímica, Instituto de Investigaciones en Bacteriología y Virología Molecular (IBaViM), Buenos Aires C1113, Argentina; gespinosa@uba.ar (F.G.-E.);; 2 Consejo Nacional de Investigaciones Científicas y Técnicas (CONICET), Buenos Aires C1425, Argentina; 3 Hospital Interzonal General de Agudos Luisa Cravena de Gandulfo, Lomas de Zamora B1832, Argentina; 4 Unidad Operativa Centro Nacional de Genómica y Bioinformática (UOCNGyB), ANLIS Dr. Carlos G. Malbrán, Buenos Aires C1282, Argentina; 5 Hospital General de Agudos Donación Francisco Santojanni, Buenos Aires C1408, Argentina

**Keywords:** carbapenem-resistance, *Enterobacterales*, *Klebsiella pneumoniae*, ST258, ST307, ST11, DTR, XDR, Argentina, Buenos Aires

## Abstract

**Background**: Carbapenem-resistant *Enterobacterales* (CRE) are a global public health concern, with carbapenem-resistant *Klebsiella pneumoniae* (CR-Kp) recognised as the highest-priority pathogen. This study aimed to investigate the epidemiological features of CRE isolates throughout the COVID-19 pandemic in Buenos Aires, Argentina. **Methods**: A prospective study was conducted in two hospitals from 2019 to 2022, recovering all CRE from inpatients. Antimicrobial susceptibility was performed by automated and/or manual tests, according to CLSI. β-lactamases detection was performed using Multiplex PCR and MALDI-TOF MS. Kp typing was assessed by multiplex PCR and/or MLST based on WGS. **Results**: 22% (359/1594) were CRE, predominantly CR-Kp. Overall, high non-susceptibility (NS) rates were observed in both centres. NS remained largely stable in HA, except for a significant increase in colistin NS, whereas HB showed a rise in NS to multiple antimicrobials over time. A significant shift from multidrug-resistant to extensively drug-resistant and difficult-to-treat phenotypes was observed across the study periods. Out of 359 CRE, *bla*_KPC_ was confirmed in 141, *bla*_NDM_ in 170, and *bla*_KPC_ + *bla*_NDM_ in 20 isolates. Before the COVID-19 pandemic, KPC was the main carbapenemase in HB, while NDM was already the prevalent one in HA. In 2022, both enzymes showed similar prevalence. *bla*_KPC-2_ and *bla*_NDM-5_ were the prevalent alleles in *K. pneumoniae*. Before the COVID-19 pandemic, *K. pneumoniae* epidemiology varied by hospital, characterised by clonal diversity; however, in 2022, CG258-*tonB79* drove the epidemiology in both hospitals. **Conclusions:** A more extensive resistance phenotype among CRE was evidenced throughout the COVID-19 pandemic, driven by carbapenemase-producing *K. pneumoniae*. NDM-5 and KPC-2 were the main carbapenemases identified. A temporal shift in carbapenemase prevalence was observed in each hospital, converging in similar frequencies of KPC and NDM by 2022 across both centres. This scenario was driven by the active dissemination of *K. pneumoniae* ST258.

## 1. Introduction

Carbapenems are broad-spectrum β-lactam antibiotics widely used for the treatment of multidrug-resistant (MDR) Gram-negative infections. Representative agents include imipenem, meropenem, doripenem, and ertapenem, all of which are extensively used worldwide [1]. Their clinical use expanded considerably following the global dissemination of extended-spectrum β-lactamase (ESBL)-producing bacteria, which hydrolyse most β-lactam antibiotics except carbapenems. However, the increased use of these agents has been accompanied by a marked rise in bacterial isolates producing carbapenemases (β-lactamases capable of hydrolysing carbapenems). As a consequence, carbapenem-resistant *Enterobacterales* (CRE), defined by resistance to at least one carbapenem and/or by carbapenemase production, have emerged as a major global health threat [1,2]. CRE have been designated as critical priority pathogens by the World Health Organisation (WHO), representing the highest level of concern for public health. In the updated WHO bacterial priority pathogens list published in 2024, carbapenem-resistant *Klebsiella pneumoniae* was ranked as the highest-priority pathogen worldwide [3]. According to the national surveillance programme, in Argentina, 36% of *K. pneumoniae* isolates recovered from inpatients in 2024 (n: 6922) were resistant to carbapenems (https://antimicrobianos.com.ar/2025/10/tablero_ram/, accessed on 15 December 2025). In our region, the predominant mechanism of carbapenem resistance among CRE is the production of acquired carbapenemases, mainly *Klebsiella pneumoniae* carbapenemase (KPC) and New Delhi metallo-β-lactamase (NDM) [4]. According to the Ambler molecular classification of β-lactamases, the carbapenemases most frequently reported worldwide belong to classes A, B, and D. Class A (e.g., KPC) and class D (e.g., OXA-48-*like*) enzymes are serine carbapenemases, whereas class B enzymes, such as NDM, are metallo-β-lactamases (MBL) that require zinc ions for catalytic activity [5]. KPC hydrolyses cephalosporins, monobactams, and carbapenems and is not inhibited by clavulanate or tazobactam; however, it is effectively inhibited by newer β-lactamase inhibitors such as avibactam, relebactam, and vaborbactam [6]. KPC is the main worldwide distributed carbapenemase, and to date, there are 284 KPC variants described in the β-lactamase database [7,8]. However, KPC-2 and KPC-3 remain the most prevalent. The global expansion of KPC-2/-3 was mediated by the presence of their coding gene in the transposon *Tn4401*, which was located in the successful *K. pneumoniae* sequence type 258 (ST258). In Argentina, KPC-2-producing *K. pneumoniae* was established in 2010 and achieved epidemic status in many settings, associated with this pandemic clone. *K. pneumoniae* ST258 remained prevalent over the following five years, when a change in its epidemiology was described [9,10]. Its almost absolute prevalence was replaced by the emergence and dissemination of more virulent lineages such as ST25 and ST11, and the worrisome high-risk clone ST307. In this country, KPC-3-*K. pneumoniae*-ST307 was first detected in 2017 [10].

Meanwhile, NDM is capable of hydrolysing almost all β-lactams except aztreonam. More than ninety variants of NDM have been described, with NDM-1 followed by NDM-5 being the most prevalent worldwide in *Enterobacterales* [8]. NDM-producing CRE are commonly resistant to almost all antimicrobial agents because of the co-occurrence of additional resistance mechanisms [11]. *bla*_NDM-1_ was detected in different plasmid replicons (IncFII, IncL/M, IncN, IncR, IncHIB-M/FIB-M, IncX3), and was rarely found to be chromosomally integrated [12]. In Argentina, NDM-1 was first detected in *Providencia rettgeri* in 2014, and this marker remained sporadically reported in other *Enterobacterales* [13,14]. In 2018, the emergence and clonal expansion of *K. pneumoniae* ST307 isolates co-producing NDM-1 and KPC-3, reported in one hospital of Buenos Aires, alerted of a worrisome scenario, regarding the remarkable features of *K. pneumoniae* ST307 and its resistance profile to ceftazidime/avibactam [14]. On the other hand, NDM-5 was first described in 2021 in *Escherichia coli* clonal complex 354 recovered from an inpatient in another hospital in Buenos Aires [15].

OXA-48-type enzyme is a carbapenem-hydrolysing class D β-lactamase first described in 2001 in a *K. pneumoniae* isolate in Turkey [16]. Up to now, more than 80 OXA-48-*like* variants have been reported in clinical *Enterobacterales* isolates worldwide [8]. OXA-48 hydrolyses penicillins at a high level and carbapenems at a low level; this enzyme shows very weak activity against expanded-spectrum cephalosporins [17]. In Argentina, carbapenem-hydrolysing OXA-48-*like* enzymes are sporadically reported; OXA-438 was detected in 2014 in an *E. coli* isolate, and later, OXA-232 was detected in 2019 in an *E. coli* ST744 isolate that co-produced MCR-1 [18,19].

Oftentimes, CRE are frequently classified as difficult-to-treat (DTR) pathogens, as they exhibit resistance to first-line therapeutic options currently available in our country, including carbapenems, β-lactam/β-lactamase inhibitor combinations, and fluoroquinolones [20]. The co-production of carbapenemases, particularly KPC and NDM, constitutes an emerging and worrisome scenario, as it compromises the efficacy of novel β-lactam/β-lactamase inhibitor combinations and further narrows therapeutic alternatives [21]. In this context, cefiderocol, a siderophore cephalosporin active against carbapenem-resistant Gram-negative pathogens, has emerged as a valuable alternative for the treatment of severe infections with limited therapeutic options [22].

This study aimed to investigate and to comparatively analyse the epidemiological features of CRE isolates recovered throughout the COVID-19 pandemic in two hospitals of the Buenos Aires Metropolitan Area, Argentina.

## 2. Results

### 2.1. Antimicrobial Susceptibilities

During the prospective study, a total of 1594 *Enterobacterales* were isolated, with 359 (22%) resistant to at least one carbapenem (CRE) (95 CRE were recovered in HA and 264 CRE in HB). The proportion of CRE recovered in HA was: 13/252, 21/257 and 61/233, in 2019, 2021, and 2022, respectively. A significant increase in CRE was detected comparing pre (5% CRE) and post-pandemic (26% CRE) periods (*p*-value < 0.0001). Meanwhile, the proportion in HB was 72/128, 55/120, 67/284, and 70/320 in 2019, 2020, 2021 and 2022, respectively. Conversely, a significant decrease in CRE was observed in HB (*p*-value < 0.0001).

*K. pneumoniae* was the prevalent species of CRE (74%) in both hospitals in all periods, followed by *Providencia stuartii* (12%) and *Escherichia coli* (4%) (Table 1 and Table S1). Blood (42%) and urine (33%) were the most common sample types for CRE recovery across all periods in HA and HB. CRE were less frequently recovered from respiratory samples (9%) (Table S1).

**Figure 1:**
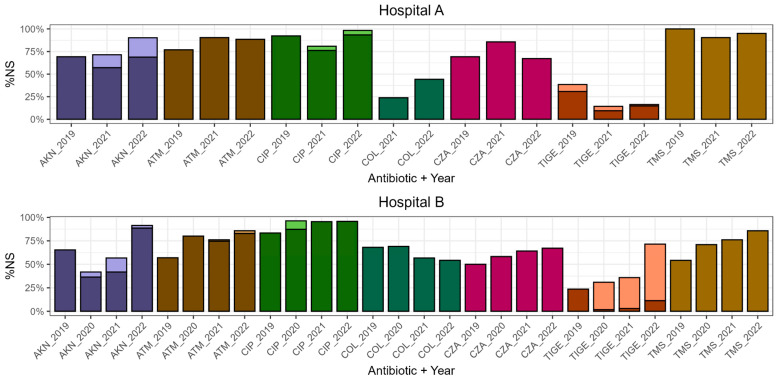
Accompanying resistance in carbapenem resistant-*Enterobacterales* isolates by hospital and year. For each antibiotic, dark colour indicates resistant isolates, and light colour indicates intermediate isolates. AKN: amikacin. ATM: aztreonam. CIP: ciprofloxacin. COL: colistin. CZA: ceftazidime/avibactam. TIGE: tigecycline. TMS: trimethoprim-sulfamethoxazole. %NS: percentage of non-susceptible isolates. Colistin rate for 2019 in HA was 0%.

Non-susceptibility (NS) rates to aztreonam, ceftazidime/avibactam (CZA), ciprofloxacin, amikacin, trimethoprim-sulfamethoxazole (TMS), tigecycline, and colistin among CRE isolates are presented in Figure 1.

In HA, the highest NS rates observed during the study period were 90% for aztreonam (2021), 86% for CZA (2021), 98% for ciprofloxacin (2022), 90% for amikacin (2022), 100% for TMS (2019), and 38% for tigecycline (2019), which remained stable across study periods (with no statistically significant differences). In contrast, colistin NS rates increased from 0% in 2019 to 24% in 2021 and 44% in 2022, showing a significant rise when comparing the pre- and post-COVID-19 pandemic periods (*p*-value = 0.003).

In HB, NS to aztreonam increased significantly from 57% in 2019 to 80% in 2020 (*p*-value = 0.008), a trend that persisted into the post-pandemic period. Significant increases between 2019 and 2022 were observed for CZA (50% to 67%, *p*-value = 0.0006), TMS (54% to 86%, *p*-value < 0.0001), and tigecycline (24% to 71%, *p*-value < 0.0001). For ciprofloxacin, NS rose significantly between 2019 and 2020 (83% to 96%, *p*-value = 0.002). Amikacin NS ranged from 42% in 2020 to 91% in 2022, representing a significant increase over time (*p*-value = 0.0002). In contrast, colistin NS rates ranged from 54% (2022) to 69% (2020), with no significant variation across study periods.

Given the resistance profiles, 82% of CRE were MDR during the COVID-19 pre-pandemic period, while the remaining 18% were XDR. Following the COVID-19 pandemic, XDR isolates increased significantly, reaching up to 88% (*p*-value < 0.0001). Additionally, in 2019, 56% of CRE were DTR, all of which were *K. pneumoniae*. DTR isolates increased significantly in 2022, up to 84% (*p*-value < 0.0001) (Table S1).

**Figure 2:**
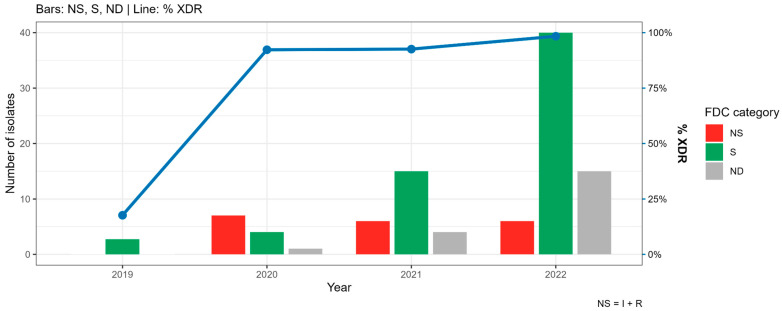
Cefiderocol (FDC) susceptibility profiles in XDR NDM-producing *K. pneumoniae*. NS: non-susceptible (resistant + intermediate), S: susceptible, ND: non-determined.

Regarding the evolution of antimicrobial resistance across the COVID-19 pandemic periods in the main pathogen, *K. pneumoniae*, a significant increase in NS isolates was observed in HA only for colistin (0% in 2019 vs. 46% in 2022, respectively, *p*-value = 0.0044). In comparison, in HB were detected a significant increase in NS isolates to amikacin (53% in 2019 vs. 91% in 2022, *p*-value < 0.0001), aztreonam (82% in 2019 vs. 100% in 2020–2022, *p*-value = 0.0008), CZA (27% in 2019 vs. 70–61% in 2020–2022, *p*-value = 0.0007), tigecycline (0% in 2019 vs. 17–69% in 2020–2022, *p*-value < 0.0001), and TMS (65% in 2019 vs. 87–94% in 2020–2022, *p*-value = 0.0003) (Table S1). With respect to cefiderocol activity, 76% of XDR MBL-producing *K. pneumoniae* were susceptible, while 15% and 9% were resistant and intermediate, respectively (Figure 2).

### 2.2. Detection of Resistance Markers

The double-disc synergy test was positive for KPC in 150/359 CRE; however, *bla*_KPC_ was confirmed in 161/359 isolates. Among 161 positive *bla*_KPC_ CRE, 20 co-harboured *bla*_NDM_. A total of 190 CRE displayed a positive double-disc synergy test for MBL, according to the detection of *bla*_NDM_, including those 20 co-producers (Table 1).

Twenty-eight CRE (negative for *bla*_KPC_ and/or *bla*_NDM_) were positive for *bla*_OXA-48-*like*_; among these, 15/28 CRE were resistant to ertapenem but not to imipenem and/or meropenem. These CRE were positive for OXA-163 by LFIA (Table S1).

Moreover, *bla*_CTX-M_ was the only ESBL coding gene detected by PCR among CRE (163/359), since *bla*_PER-2_ was not present in any of them. In turn, *bla*_CMY_ was found in 48 CRE and *bla*_CTX-M_ + *bla*_CMY_ in 18. The co-carriage of *bla*_KPC_ + *bla*_CTX-M_ was detected in 54 CRE, *bla*_KPC_ + *bla*_CMY_ in 1, *bla*_NDM_ + *bla*_CTX-M_ in 75, *bla*_NDM_ + *bla*_CMY_ in 38, while *bla*_KPC_ + *bla*_NDM_ + *bla*_CTX-M_ was detected in 7 CRE, and *bla*_KPC_ + *bla*_NDM_ + *bla*_CTX-M_ + *bla*_CMY_ in 3 (Table S1).

**Figure 3:**
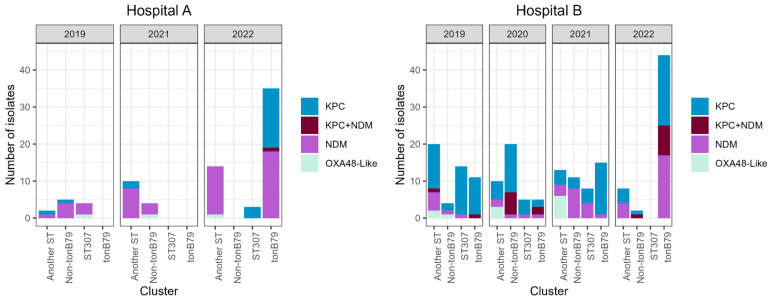
Molecular typing of carbapenemase-producing *K. pneumoniae*.

Among *K. pneumoniae* isolates, a high prevalence of *bla*_KPC-2_ with respect to *bla*_KPC-3_ was observed. Accordingly, specific peaks for KPC-2 and KPC-3 were detected by MALDI-TOF MS (87% corresponded to KPC-2 and 13% to KPC-3). *bla*_NDM-5_ was the most frequent variant, followed by *bla*_NDM-1_ (77% and 23%, respectively) (Table S1). Before the COVID-19 pandemic, KPC was the main carbapenemase in HB, while NDM was already the prevalent one in HA. In 2022, both enzymes showed similar prevalence (Figure 3).

### 2.3. Molecular Typing of K. pneumoniae Isolates

In HA, among the 77 *K. pneumoniae* isolates, 35 corresponded to the CG258-*tonB79* cluster, 9 to the CG258-non-*tonB79* cluster, 7 belonged to ST307, and 26 to other STs (Table S1). The emergence and widespread expansion of CG258-*tonB79* was observed between 2019 and 2022; in parallel, an increase in *bla*_KPC_ relative to *bla*_NDM_ was detected. During 2022, the first KPC + NDM-producing isolate was recovered from this hospital (Figure 3).

In HB, among the 190 *K. pneumoniae* isolates, 75 corresponded to the CG258-*tonB79* cluster, 37 to the CG258-non-*tonB79* cluster, 27 belonged to ST307, and 51 to other STs (Table S1). The clusters CG258-*tonB79* and ST307 were present in all periods except 2022, when ST307 was absent. Also, in 2022, CG258-*tonB79* significantly increased its prevalence (fourfold) with respect to 2019 (*p*-value < 0.0001). CG258-non-*tonB79* cluster displayed its greatest prevalence during 2020. Unlike HA, *bla*_KPC_ was the prevalent carbapenemase persisting over the years in all clusters. Another difference with respect to HA is the presence of *bla*_KPC_ + *bla*_NDM_ co-harbouring isolates since 2019 in both CG258 clusters. In this hospital, during 2022, an increase in *bla*_NDM_ detection was observed, associated with the expansion of CG258-*tonB79* (Figure 3).

### 2.4. MLST of NDM-Producing K. pneumoniae Isolates

Out of 103 isolates subjected to WGS, all genome assemblies passed the quality control proposed by the pipeline, with no contamination markers detected. Coverage ranged from 28× to 809×, genome size from 5.3 Mb to 6 Mb and number of contigs from 35 to 206 (Table S1).

WGS confirmed ST258 as the unique ST in the prevalent CG258-*tonB79* cluster and ST11 as the only lineage in the CG258-non-*tonB79* cluster. In addition, WGS showed that isolates typed as ST307 by PCR actually corresponded to ST307 and also ST5994. Seventy-one out of 103 isolates corresponded to epidemic lineages: ST258 (n:41), ST11 (n:20), and ST307 (n:10). These results showed good correlation with PCR typing. The remaining 32 isolates corresponded to ST107 (n:6), ST1788 (n:4), ST1805 (n:3), ST207 (n:3), ST3430 (n:3), ST5994 (n:3), ST1229 (n:2), ST525 (n:2), ST15 (n:1), ST294 (n:1), ST45 (n:1), ST485 (n:1), ST6148 (n:1) and ST870 (n:1) (Table 2).

Among epidemic lineages, *bla*_NDM-5_ was confirmed as the prevalent allele being detected in 18, 16, and 9 isolates of ST258, ST11, and ST307, respectively. The second prevalent *bla*_MBL_ was *bla*_NDM-1_, which was present in 15, 4, and 1 isolates of ST258, ST11, and ST307, respectively. Even *bla*_NDM-5_ was detected in 30 isolates belonging to different STs (Table 2).

## 3. Discussion

This study provides a dynamic view of the epidemiology, resistance profiles, and molecular characteristics of CRE recovered in two hospitals over four years spanning the COVID-19 pandemic. Across both hospitals and all study periods, *K. pneumoniae* was the predominant CRE in line with the well-established role of *K. pneumoniae* as the main driver of carbapenem resistance worldwide [3]. An increase in more extensive resistance phenotypes was observed; while MDR isolates predominated before the COVID-19 pandemic, the post-pandemic period was characterised by a striking increase in XDR isolates. Similarly, DTR phenotypes increased, particularly among *K. pneumoniae*, further limiting therapeutic options. This concomitant increase in XDR and DTR isolates indicates a progressive narrowing of therapeutic options, expanded to last-line agents. Overall, the year-by-year analysis revealed distinct resistance trajectories between hospitals. While HA showed relative stability in most NS rates, except for a marked increase in colistin resistance, HB exhibited a progressive and sustained rise in NS to multiple antimicrobial classes, suggesting heterogeneous local dynamics. The observed increase in XDR phenotypes may be related to the antimicrobial consumption. Consistent with the resistance patterns detected in CRE, penicillins and their derivatives were the most consumed antibiotic class from 2019 to 2022 in Argentina (46.5–51.6% annually). Although quinolones ranked third in 2020 and 2021 (10.9% and 11.3%, respectively), by 2022 they were surpassed by other β-lactams, including cephalosporins, monobactams, and carbapenems, while penicillins remained the most widely used class [23].

A significant rise in CRE was observed in HA when comparing the pre- and post-pandemic periods, consistent with reports from other regions describing the impact of the COVID-19 pandemic on antimicrobial resistance [24]. Notably, other regional studies have reported significant shifts in antimicrobial resistance profiles and antibiotic consumption during the COVID-19 pandemic compared with the pre-pandemic period [25,26,27]. The divergent resistance dynamics in HA with respect to HB underscore the importance of hospital-specific surveillance rather than relying on aggregated regional data.

NDM was detected across the different *Enterobacterales* species, with NDM-5 being the prevalent variant in *K. pneumoniae*. The second most common carbapenemase was KPC, mainly KPC-2, which was infrequent in species other than *K. pneumoniae*. This pattern is consistent with regional and global trends [4,24,28]. A temporal shift in carbapenemase prevalence was observed: while KPC predominated in HB before the pandemic and NDM was already prevalent in HA, both enzymes reached similar frequencies by 2022. This convergence suggests ongoing dissemination and replacement dynamics that may be driven by clonal expansion rather than sporadic horizontal gene transfer alone. The increase in NDM-producing isolates correlated with CZA resistance, and with aztreonam resistance when associated with CTX-M. Although CZA was introduced in Argentina as a targeted therapeutic option against KPC-producing *Enterobacterales*, its clinical implementation may have exerted selective pressure favouring the emergence and dissemination of NDM-producing CRE.

Double carbapenemase-producing isolates had been detected only once in our country before the studied period (2019), where KPC-3 + NDM-1-producing *K. pneumoniae* ST307 was detected in HB [14]. At the end of the COVID-19 pandemic, an increase in this resistance profile was observed in HB; however, it was due to the expansion of KPC-2 + NDM-5-producing *K. pneumoniae* ST258. All but one double carbapenemase producers were confined to CG258 (Table S1). The discrepancy between KPC detection based on phenotypic synergy tests with respect to LFIA and/or molecular methods could be explained by the presence of isolates co-harbouring *bla*_KPC_ + *bla*_NDM_. In these co-producing isolates, inhibition of KPC by phenylboronic acid was not observed, likely due to the concurrent hydrolytic activity of NDM masking the KPC inhibition.

Since 2022, *K. pneumoniae* epidemiology has been driven by ST258, associated with the carriage of *bla*_KPC-2_ and/or *bla*_NDM-5_, in both HA and HB. Also, other epidemic/high-risk clones were confirmed, such as ST11 and ST307. As well as the ST15, ST45, and ST485, previously found in Argentina and others that have never been reported before in this country, were evidenced [10,29]. The detection of *bla*_NDM-5_ in a wide variety of STs highlights the successful dissemination of its genetic platform beyond classical high-risk clones [30].

Susceptibility testing of cefiderocol by broth microdilution requires iron-depleted Mueller–Hinton broth, whose multi-step preparation renders the method labour-intensive, difficult to standardise, and associated with reduced reproducibility, thereby limiting its routine implementation in clinical laboratories [31]. In addition, endpoint interpretation can be difficult and prone to error due to in vitro visual artefacts, as noted in the M100 manual of the Clinical and Laboratory Standards Institute (CLSI) [32], requiring further standardisation across successive M100 editions. Despite the marked increase in XDR *K. pneumoniae* isolates, cefiderocol retained activity against most XDR NDM-producing strains. However, resistant isolates were detected. Given that cefiderocol is not currently used in clinical practice in Argentina, this resistance may have arisen through indirect selective pressures. The high prevalence of NDM producers in our setting warrants particular attention, as NDM may act as a proxy for the emergence of cefiderocol resistance through the co-expression of additional resistance mechanisms. Moreover, overexpression of the *bla*_NDM_ has been linked to the in vivo development of cefiderocol resistance during treatment [22,31]. These observations underscore the need for continuous surveillance of cefiderocol activity, especially in regions with endemic dissemination of NDM-producing *Enterobacterales*.

The limitations of this study include the following: Isolates from HA were unavailable during 2020, limiting direct comparison with the early COVID-19 pandemic period. Whole-genome sequencing was restricted to MBL-producing *K. pneumoniae*, while other isolates were characterised by PCR-based methods, and non-*K. pneumoniae* species were not molecularly typed. In addition, bioinformatic analyses did not detail outbreak or plasmid investigations. Despite these limitations, our results provide a valuable genomic baseline for future studies addressing the evolution and dissemination of high-risk clones in our region.

## 4. Materials and Methods

### 4.1. Bacterial Isolates, Antibiotic Susceptibility and Categorization

A prospective, observational, and multicentric study was carried out in two public hospitals (HA and HB) during May-September in 2019 (COVID-19 pre-pandemic), 2020–2021 (COVID-19 pandemic), and 2022 (COVID-19 post-pandemic). HA is a second-level hospital containing 200 beds, while HB is a third-level hospital containing 300 beds. Both hospitals experience substantial demand by socioeconomically vulnerable patients in densely populated peripheral areas of Buenos Aires metropolitan area, for whom the public healthcare system represents the main point of access to medical care.

In the hospitals, bacterial isolation from clinical specimens was carried out in accordance with the procedures outlined in the Manual of Clinical Microbiology of the American Society for Microbiology [33]. Antibiotic susceptibilities to aminopenicillins, cephalosporins, monobactams, carbapenems, ciprofloxacin, gentamicin, amikacin, tigecycline, and TMS were determined using automated methods (Phoenix, Bruker Daltonics, Bremen, Germany, in HA and Vitek2, bioMérieux, Craponne, France and BD in HB). CZA and colistin susceptibilities were determined by diffusion test and col-drop test, respectively. Interpretation was performed according to the Clinical and Laboratory Standards Institute (CLSI, https://clsi.org/) breakpoints applicable for each study year, except for ceftazidime–avibactam (CZA) and tigecycline, for which the European Committee on Antimicrobial Susceptibility Testing (EUCAST, https://www.eucast.org/) and U.S. Food and Drug Administration (FDA) criteria were applied [32,34,35,36]. All *Enterobacterales* isolates resistant to at least one carbapenem (meropenem, imipenem, and/or ertapenem) were delivered to IBaViM Institute in an encrypted way (to preserve the identity of the patients). HA did not conserve isolates to be delivered in 2020 due to health system overload. CRE identification was controlled at IBaViM by MALDI-TOF MS using a Microflex LT mass spectrometer (Bruker Daltonics, Bremen, Germany) with flexControl 3.4 software (Bruker Daltonics, Bremen, Germany), using the in situ extraction method, which consists of adding 1 μL of formic acid before sealing with the commercial hidroxicianocinamicacid (HCCA) matrix. All isolates were stored at −70 °C in brain heart infusion broth with 20% glycerol.

CRE isolates were classified as Multi-resistant (MDR), Extremely resistant (XDR), and Pan-resistant (PDR), according to Magiorakos et al., 2012 [37]. Intrinsic resistance was not addressed for MDR, XDR and PDR definitions. In accordance with the antimicrobial categories listed for *Enterobacteriaceae*, the MDR was assumed when an isolate was non-susceptible to at least 1 agent in ≥3 antimicrobial categories. XDR was assumed when the isolate was non-susceptible to at least 1 agent in all but 2 or fewer antimicrobial categories, and PDR was assumed for non-susceptibility to all agents in all antimicrobial categories for each isolate. Additionally, susceptibility to cefiderocol (FDC) was determined in 81 MBL-producing *K. pneumoniae* isolates, categorised as XDR according to Magiorakos et al.

In addition, the isolates were categorised as DTR pathogens, following the practical proposal of Kadri et al. [20] when resistance to all first-line agents was observed, such as resistance to all β-lactams, including carbapenems and β-lactamase inhibitor combinations, and fluoroquinolones.

### 4.2. Phenotypic Detection of Carbapenemases

The presence of carbapenemases was analysed by double disc inhibition test using both phenylboronic acid (PBA) (300 µg) and EDTA (1 µmol) for the detection of KPC and/or metallo-β-lactamase (MBL), as well as lateral flow immunoassay (LFIA) (RESIST-3 O.O.K K-SET, Britania, Argentina) to differentiate among KPC, OXA-48, and OXA-163. The direct detection of specific β-lactamases, such as KPC-2, KPC-3, KPC-8, and KPC-31, was carried out by analysing the protein spectrum obtained by MALDI-TOF MS, as described by Espinosa et al. in *K. pneumoniae* [38,39].

### 4.3. Genotypic Detection of β-lactamases Coding Genes

The carbapenemase-encoding genes *bla*_KPC_, *bla*_OXA-48-*like*_, *bla*_NDM_, *bla*_IMP_, and *bla*_VIM_ were screened by multiplex PCR using total DNA as template obtained by the boiling method [40,41]. In addition, *bla*_CTX-M_, *bla*_CMY_, and *bla*_PER-2_ were screened by Multiplex-PCR using the primers CTX-MU-1, CTX-MU-2, PER-2-PLUS, PER-2-MINUS, CIT-MF and CIT-MR. In *K. pneumoniae*, larger amplicons of carbapenemase coding genes were obtained using the specific primers KPC-F and KPC-R for *bla*_KPC_, and NDM-F and NDM-R for *bla*_NDM_. (Table 3). Amplicons were sequenced at external facilities and analysed using the BLAST tool of the BLDB database (http://www.bldb.eu:4567/, accessed on 1 March 2025) [8].

### 4.4. Molecular Typing of K. pneumoniae Isolates

The two clusters of *K. pneumoniae* CG258: CG258-*tonB79* (e.g., ST258, ST379, ST418, and ST512) and CG258-non-*tonB79* (e.g., ST11, ST340, and ST437) were investigated by Multiplex-PCR proposed by Yu et al. (Table 3) [42]. In parallel, isolates belonging to ST307 were characterised by simplex PCR following Lowe et al. (Table 3) [43].

**Table 3:** Primers used in this study.

Gene	Primer	Sequence 5′-3′	Product Size (bp)	Reference	Purpose
*bla*_KPC_	KPCm-F	CGTCTAGTTCTGCTGTCTTG	798	Poirel et al. (2011) [40]	β-lactamase detection
	KPCm-R	CTTGTCATCCTTGTTAGGCG			
	KPC-F ^b^	ATGTCACTGTATCGCCGTC	890	Bradford et al. (2004) [44]	
	KPC-R ^b^	TTTTCAGAGCCTTACTGCCC			
*bla*_NDM_	NDM-F ^a^	GGTTTGGCGATCTGGTTTTC	621	Poirel et al. (2011) [40]	
	NDM-R ^a^	CGGAATGGCTCATCACGATC			
*bla*_OXA48-like_	OXAm-F	GCGTGGTTAAGGATGAACAC	438	Poirel et al. (2011) [40]	
	OXAm-R	CATCAAGTTCAACCCAACCG			
*bla*_VIM_	VIM-F	GATGGTGTTTGGTCGCATA	390	Poirel et al. (2011) [40]	
	VIM-R	CGAATGCGCAGCACCAG			
*bla*_IMP_	IMP-F	GGAATAGAGTGGCTTAAYTCTC	232	Poirel et al. (2011) [40]	
	IMP-R	GGTTTAAYAAAACAACCACC			
*bla*_SPM_	SPM-F	AAAATCTGGGTACGCAAACG	271	Poirel et al. (2011) [40]	
	SPM-R	ACATTATCCGCTGGAACAGG			
*bla*_CMY_	CIT-MF	TGGCCAGAACTGACAGGCAAA	462	Perez-Perez et al. (2002) [45]	
	CIT-MR	TTTCTCCTGAACGTGGCTGGC			
*bla*_CTX-M_	CTX-MU-1	ATG TGC AGY ACC AGT AAR GT	593	Pagani et al. (2003) [46]	
	CTX-MU-2	TGG GTR AAR TAR GTS ACC AGA			
*bla*_PER-2_	PER2 PLUS	GTAGTATCAGCCCAATCCCC	739	Pasteran et al. (2006) [47]	
	PER 2 MINUS	CCAATAAAGGCCGTCCATCA			
*pilV*	pilV-F3	CGATGGCGCTGGCGACGATTAT	627	Yu et al. (2018) [42]	*K. pneumoniae* typing
	pilV-R3	CCCGATGGGCAAGAACATGCGT			
*Kphp*	kphp-F3	TGGCGGGTAATGCCCGATCAGT	236	Yu et al. (2018) [42]	
	kphp-R3	AGGCCGCTTTCCATAAGCCGTT			
*pilL*	pilL-F2	CGGTATTTGCTCTGCGTGATAG	350	Yu et al. (2018) [42]	
	pilL-R2	TGGTTATACAGAACGGCATTGG			
*Khe*	Khe-F3	CCGGAGCGTTTTTCAATCGGCG	441	Yu et al. (2018) [42]	
	Khe-R3	CGCTTCGCCCCTCACCTGAAAT			
*72_phage2 * ^c^	ST-307-F	AGGAAAGTCGCCGCAGGAGGAT	628	Lowe et al. (2011) [43]	
	ST-307-R	TGCTGCTGCCATAAAACGCACCT			

^a^ Primers used for both screening and sequencing of *bla*_NDM_ genes. ^b^ Primers used for sequencing of *bla*_KPC_ genes. ^c^ Primers used for the detection of *K. pneumoniae* ST307.

Additionally, Multi Locus Sequence Typing (MLST) was assessed in 103 NDM-producing *K. pneumoniae* genomes using the BIGSdb-Pasteur database (https://bigsdb.pasteur.fr/klebsiella/, accessed on 1 October 2025) [48,49]. Genomic DNA was extracted using a commercial kit (DNA Puriprep B-Kit, Inbio Highway, Argentina) and sequenced (WGS) by Illumina Miseq. Raw reads were processed through a standardised bioinformatics pipeline (NIHR Global Health Research Unit De novo assembly pipeline). Adapter sequences and low-quality bases were removed using Trimmomatic (v0.38), followed by read error correction with Lighter (v1.1.1). To normalise sequencing depth, reads were downsampled to 100× coverage using SeqTK (v1.3), and overlapping paired-end reads were merged with FLASH (v1.2.11). De novo genome assembly was performed using SPAdes (v3.12.0). Quality control of raw and processed data was assessed using FastQC (v0.11.8), MultiQC (v1.7), and Qualifyr (v1.4.4). Species identification was conducted with Bactinspector (v0.1.3), and potential sample contamination was evaluated using Confindr (v0.7.2) [50].

### 4.5. Statistical Analysis

Categorical variables, including non-susceptibility (NS) rates, resistance phenotypes (MDR/XDR/DTR), prevalence of resistance genes, and clonal distribution across hospitals and study periods, were compared using Fisher’s exact test. This test was selected due to our sample sizes. A *p*-value < 0.05 was considered statistically significant. The R software (version 4.5.1) has been used to analyse and graph the data [51].

## 5. Conclusions

This study evidences the increase in more extensive resistance phenotypes among CRE throughout the COVID-19 pandemic, driven by carbapenemase-producing *K. pneumoniae*, accompanied by a shift toward XDR and DTR phenotypes. Resistance dynamics differed between centres: while HA remained relatively stable—except for a rise in colistin resistance—HB showed a sustained increase in non-susceptibility across multiple antimicrobials. This trend may reflect local antimicrobial consumption patterns, which could contributed to the selection of more resistant isolates in Argentina during the study period.

NDM-5 and KPC-2 were the main carbapenemases identified, in line with regional and global trends. A temporal shift in carbapenemase prevalence was observed in each hospital, converging in similar frequencies of KPC and NDM by 2022 across both centres. This scenario was driven by the active dissemination of *K. pneumoniae* ST258.

## Figures and Tables

**Table 1:** Distribution of β-lactamase coding genes by species.

Bacterial Species	*bla*_KPC_	*bla*_KPC_ + *bla*_NDM_	*bla*_NDM_	*bla*_OXA-48-_ *_like_*	*bla*_CTX-M_	*bla*_CTX-M_ + *bla*_CMY_	*bla*_CMY_
*K. pneumoniae* (n: 267)	133	20	99	15	135	16	12
*Providencia stuartii* (n: 45)	0	0	37	8	2	0	23
*Escherichia coli* (n: 14)	1	0	10	3	2	0	2
*Enterobacter cloacae* (n: 9)	4	0	5	0	2	0	3
*Proteus mirabilis* (n: 9)	0	0	8	1	0	0	5
*Klebsiella oxytoca* (n: 5)	1	0	4	0	2	1	0
*Citrobacter freundii* (n: 3)	1	0	2	0	1	1	1
*Klebsiella aerogenes* (n: 2)	0	0	2	0	1	0	1
*Morganella morganii* (n: 1)	0	0	1	0	0	0	0
*Proteus penneri* (n: 1)	0	0	1	0	0	0	1
*Providencia rustigianii* (n: 1)	0	0	0	1	0	0	0
*Salmonella* spp. (n: 1)	1	0	0	0	0	0	0
*Serratia marcescens* (n: 1)	0	0	1	0	0	0	0
n total: 359	141	20	170	28	145	18	48

**Table 2:** STs distribution among NDM-producing *K. pneumoniae*.

MLST	PCR Typing	*bla*_KPC-2_ + *bla*_NDM-1_	*bla*_KPC-2_ + *bla*_NDM-5_	*bla*_NDM-1_	*bla*_NDM-5_
ST258 (n: 41)	CG258 *ton79* cluster	4	8	11	18
ST11 (n: 20)	CG258 non-*ton79* cluster	3	0	1	16
ST307 (n: 10)	ST307	0	0	1	9
ST107 (n: 6)	other ST	0	0	0	6
ST1788 (n: 4)	other ST	0	0	0	4
ST1805 (n: 3)	other ST	0	0	0	3
ST273 (n: 3)	other ST	0	0	0	3
ST3430 (n: 3)	other ST	0	0	0	3
ST5994 (n: 3)	ST307	0	0	0	3
ST1229 (n: 2)	other ST	0	0	0	2
ST525 (n: 2)	other ST	0	0	0	2
ST15 (n: 1)	other ST	0	0	0	1
ST294 (n: 1)	other ST	0	0	0	1
ST45 (n: 1)	other ST	0	0	0	1
ST485 (n: 1)	other ST	0	0	0	1
ST6148 (n: 1)	other ST	0	0	1	0
ST870 (n: 1)	other ST	0	0	1	0
Total (n: 103)		7	8	15	73

## Data Availability

The data will be available upon request.

## Supplementary Materials

The following supporting information can be downloaded at: https://www.mdpi.com/article/10.3390/antibiotics15030273/s1, Table S1: Isolates metadata, antimicrobial susceptibility, resistance genes, typing, and genome quality metrics.

## Abbreviations

The following abbreviations are used in this manuscript:

AKN	Amikacin
ATM	Aztreonam
CG	Clonal group
CIP	Ciprofloxacin
COL	Colistin
CRE	Carbapenem-resistant *Enterobacterales*
CZA	Ceftazidime/avibactam
FDC	Cefiderocol
I	Intermediate resistance
Kp	*Klebsiella pneumoniae*
KPC	*Klebsiella pneumoniae* carbapenemase
LFIA	Lateral flow immunoassay
MBL	Metallo-β-lactamase
NDM	New Delhi metallo-β-lactamase
NS	Non-susceptible
R	Resistant
S	Susceptible
ST	Sequence type
TIGE	Tigecycline
TMS	Trimethoprim-sulfamethoxazole.
WGS	Whole genome sequencing
